# Correlation between adiponectin rs2241766 and rs266729 polymorphisms and risk of papillary thyroid cancer

**DOI:** 10.22099/mbrc.2022.43012.1714

**Published:** 2022

**Authors:** Mohsen Maleki, Mansour Karajibani, Mohsen Sarvani, Farzaneh Montazerifar, Saeedeh Salimi, Zahra Heidari6

**Affiliations:** 1 Department of Nutrition, School of Medicine, Zahedan University of Medical Sciences, Zahedan, Iran; 2 Health Promotion Research Center, Department of Nutrition, School of Medicine, Zahedan University of Medical Sciences, Zahedan, Iran; 3 Cellular and Molecular Research Center, Research Institue of Cellular and Molecular Sciences in Infectious Diseases, Zahedan University of Medical Sciences, Zahedan, Iran; 4 Department of Clinical Biochemistry, School of Medicine, Zahedan University of Medical Sciences, Zahedan, Iran; 5 Pregnancy Health Research Center, Department of Nutrition, School of Medicine, Zahedan University of Medical Sciences, Zahedan, Iran; 6 Department of Endocrinology and Metabolism, Zahedan University of Medical Sciences, Zahedan, Iran; ¥ Mansour Karajibani and Mohsen Sarvani contributed equally to the project.

**Keywords:** Adiponectin, Papillary thyroid cancer, Polymorphism

## Abstract

About 60-80% of thyroid cancer (TC) cases are papillary thyroid cancer (PTC). Studies have shown that serum adiponectin levels are inversely related to the risk of TC and PTC. Aim of the present study was to evaluate the association between adiponectin rs2241766 and rs266729 polymorphisms and risk of PTC. 122 PTC patients and 128 healthy subjects were enrolled in the study. PCR-RFLP and ARMS-PCR methods were used for genotype analysis. The rs266729 polymorphism did not correlate with risk of PTC. As regard rs2241766 polymorphism, the frequency of the GG genotype did not have a significant difference between the two groups, although, PTC cases showed higher frequency of GT genotype compared to controls (OR=2.87, 95% CI=1.56-5.28, P=0.001). We observed a significant association between adiponectin rs2241766 polymorphism and PTC, however, our result showed no significant relationship between adiponectin rs266729 polymorphism and risk of PTC.

## INTRODUCTION

Thyroid cancer (TC) is considered as the most common form of endocrine cancer and also papillary thyroid carcinoma (PTC) which is the most frequent form of well-differentiated thyroid cancer, accounts for about 60-80% of TC cases. males [1]. Although the etiology of PTC is not yet fully understood, there are several risk factors such as ionizing radiation, thyroid stimulating hormone (TSH) level and its receptors, iodine deficiency, environmental and genetic factor and body mass index (BMI) which are known as risk factors of developing the disease [2]. Obesity can be defined as BMI higher than 30 Kg/m^2^ [3, 4]. Epidemiological studies have also shown that there is an association between obesity and TC risk. [5]. Adipose tissue as highly active metabolic and endocrine organ secretes molecules called adipokines [6]. Adiponectin is an anti-apoptotic adipokine released from adipose tissue [7], encoded by the *ADIPOQ* gene consisting of three exons and two introns located on chromosome 3q27.3 [8]. Concentration of the adiponectin reduces in obesity and circulating adiponectin levels are found to be significantly associated with increased adipose tissue in adults [9]. Studies have shown that serum adiponectin level is inversely associated with the risk of TC and PTC [10]. In 2003, AdipoR1 and AdipoR2 were identified as the two adiponectin receptors. [11]. Cheng et al. (2013) demonstrated that protein levels of AdipoR1 and AdipoR2 are enhanced in some thyroid cancer. The negative expression of adiponectin receptors in PTC is associated with a higher risk of unfavorable prognosis, and a lower chance of survival [12]. 

A single nucleotide polymorphism (SNP) is considered as a DNA sequence variation occurring in people if its prevalence is more than 1% [13]. Growing body of evidence suggest the association between SNPs in the adiponectin gene and plasma levels of the adiponectin [5, 14]. The adiponectin gene has more than 160 SNPs. The rs2241766 (+ 45T/G) polymorphism in exon 2 of the *ADIPOQ* gene has been shown to create a haplotype associated with obesity and insulin resistance [15]. The rs266729 is located in the promoter region of the *ADIPOQ* gene promoter at position -11,377 and causes C to G substitution. Studies indicate the regulatory role of this polymorphism in promoter activity and plasma adiponectin levels. The association of rs266729 with high body mass index has also been reported [14]. Low serum levels of APN in obesity are associated with the development and progression of cancer, and therefore APN genetic variants can cause genetic predisposition to cancer [3].

Regarding the important role of adiponectin in developing PTC, the presents study focused on the relationship between adiponectin rs2241766 and rs266729 gene polymorphisms with the risk of PTC in Iranian patients.

## MATERIALS AND METHODS

**Study subjects: **Zahedan University of Medical Sciences' Ethics Committee approved the protocol for this case-control study (IR.ZAUMS.REC.1399.523). The study included 122 patients with PTC and 128 healthy subjects (matched for age and gender), who were referred to the endocrinology clinic at Ali-ebn Abitaleb Hospital, Zahedan, South-East Iran. In the case group, the study exclusion criteria were as follows: statin and antihypertensive drug use, a history of neck irradiation, thyroid disease, a history of thyroid surgery. In the control group, subjects with a history of diabetes Mellitus, autoimmune disease, malignancy, and renal or hepatic disorders were excluded from the study.

**DNA extraction and genotyping analysis: **500 µl of EDTA-containing blood was used for DNA extraction by the salting-out method. The quality of the DNA was checked by agarose gel electrophoresis and OD_260_/OD_280_ ratio analysis. The PCR-RFLP method was used for rs266729, as described previously [16]. Tetra-primer ARMS-PCR method was designed for rs2241766 genotyping. PRIMER1 web service was used for tetra-primer ARMS-PCR primer design [17]. The sequences of tetra-primer ARMS-PCR were as follows: forward outer: 5ʹ-CTCTGCTGAG ATGGACGGAGTCCT-3ʹ, reverse outer: 5ʹ-GAGGTCTGTGATGAAAGAGGCCAGA-3ʹ, forward inner (G allele): 5ʹ-TGTTCTACTGCTATTAGCTCTGCCCAGG-3ʹ, and reverse inner (T allele): 5ʹ-GAGTCGTGGTTTCCTGGTCACGA-3ʹ. ARMS-PCR products and digested fragments were electrophoresed on 3% agarose gel and visualized under UV illumination. For rs266729 Digested fragments sizes were: CC genotype (250 bp), CG genotype (250/138/112 bp), and GG genotype (138/112 bp). For rs2241766 PCR products sizes were: outer control (353 bp), GG genotype (233 bp), and TT genotype (170 bp). 

**Statistical analysis: **We evaluated the impact of SNPs on disease development by using the odds ratio (OR) and 95% CI. χ^2^ tests were used to analyze demographic characteristics. P<0.05 was considered statistically significant. We used SPSS version 23 for data analysis.

## RESULTS

Demographic and clinical characteristics of PTC patients and controls are shown in Table 1. With respect to age and gender, there were no significant differences between the two groups. We observed no significant difference between the two groups in terms of BMI. Table 1 also contains information about the tumor size and stages.

**Table1:** Demographic and clinical characteristics of papillary thyroid carcinoma patients and controls

**Characteristics**		**PTC n=122**	**Control n=128**	**P-value**
**Age**		36.2±12.1	34.7±10.3	0.29
**BMI**		25.9±5.5	25.5±4.2	0.51
**Gender**	Male	23(18.9)	23(18)	
	Female	99(81.1)	105(82)	0.87
				
**Location**	Right Lobe	55(45.1)		
	Left Lobe	53(43.5)		
	Both Lobes	14(11.4)		
**Tumor Size**	<1cm	16 (13.1)		
	≥1cm	96(78.8)		
	Unknown	10(8.1)		
**TNM stage**	I	67(54.9)		
	II	13(10.7)		
	III	14(11.5)		
	IV	13(10.7)		
	Unknown	15(12.2)		
**N Stage**	N0	75(61.5)		
	N1	33 (27.1)		
	Unknown	14(11.5)		
**Vascular invasion**	Positive	18(14.7)		
	Negative	87(71.3)		
	Unknown	17(13.9)		
				
**Capsular invasion**	Positive	24(19.7)		
	Negative	82(67.2)		
	Unknown	16(13.1)		
**Extrathyroidal expansion**	Positive	13(10.7)		
	Negative	93(76.2)		
	Unknown	16(13.1)		

The rs266729 and rs2241766 polymorphisms in control group were in Hardy-Weinberg equilibrium. There was no significant association between rs266729 polymorphism and risk of PTC. Regarding the rs2241766, the frequency of GT genotypes in the PTC group was significantly higher than that in the controls and may be a risk factor for PTC (OR= 2.87, 95% CI= 1.56–5.28, P=0.001) (Table 2). 

According to Table 3, the frequency of the CG haplotype in the PTC group was higher than that in controls (16.3% *vs* 9.1%), suggesting it may be a risk factor for PTC (OR=1.99, 95% CI=1.1-3.6, P=0.024). In PTC patients, the frequency of the GG haplotype was higher (5.3% *vs* 1%), but the difference was not statistically significant (P=0.061). In addition, controls had a higher frequency of the GT haplotype than PTC patients (12.2% *vs* 9.4%), but the difference was not statistically significant (P=0.76).

**Table 2:** Association between the rs266729 and rs2241766 polymorphisms and risk of PTC

**Polymorphism**	**PTC n (%)**	**Control n (%)**	**OR**	**95%CI**	**P**
**rs266729**					
**CC**	90 (74)	98 (77)	1.0		
**CG**	28 (23)	26 (20)	1.17	0.64-2.14	0.606
**GG**	4 (3)	4 (3)	1.08	0.26-4.48	0.906
					
**rs2241766**					
**TT**	75 (61)	105 (82)	1.0		
**GT**	41 (34)	20 (16)	2.87	1.56-5.28	0.001
**GG**	6 (5)	3 (2)	2.80	0.68-11.5	0.154

**Table 3:** Haplotypes frequency of the *ADIPOQ* polymorphisms in PTC patients and controls

**rs266729**	**rs2241766**	**PTC**	**Control**	**OR (95% CI)**	**P**
**C**	**T**	69.0	77.7	1.0	**-**
**C**	**G**	16.3	9.1	1.99 (1.1-3.6)	0.024
**G**	**T**	9.4	12.2	0.91 (0.51-1.62)	0.76
**G**	**G**	5.3	1.0	4.45 (0.94-21)	0.061

## DISCUSSION

From a molecular point of view, some mutations, including proto-oncogene mutations such as RAS and BRAF, are often observed in PTC [18]. Firminger and Skelton (1953) reported the first case of PTC in twins and suggested that genetic background was involved in PTC [19]. Adiponectin is one of the most important Adipocytokines secreted by adipocytes and is inversely associated with obesity. It has anti-diabetic, anti-inflammatory and anti-tumor effects. [20]. The evidence suggests that hypoadiponectinemia can increase the risk of TC [21, 22]. Thyroid tissue expresses the adiponectin receptors (ADIPO-R1, and ADIPO-R2), which has also been observed in TC tissue [23]. Our results showed that there was no significant association between rs266729 polymorphism of *ADIPOQ* and the risk of PTC development. Genetic models and allelic distribution also showed similar results. In addition to modifying the amino acid sequences, SNPs can also be silent or appear in noncoding regions. They may also affect promoter activity and mRNA stability. As a result, analysis of gene variants is effective in further understanding our changes in the incidence of disease [24]. 

To the best of our knowledge, this is the first report of the association between adiponectin rs2241766 and rs150299 gene polymorphism and risk of PTC. The results obtained from studies conducted on the effect of this polymorphism on risk of cancer are contradictory. A meta-analysis showed that rs2241766 and rs150299 polymorphisms had a protective effect on cancer incidence, while similar to our results, they did not find a significant relationship between the rs266729 and cancer incidence [25]. A meta-analysis showed a correlation between rs266729 and cancer incidence [26]. Our results showed that the rs2241766 associated with the risk of PTC. The frequency of rs2241766 GT genotype in PTC group was also higher than that in controls and it seems that this allele acts as a risk factor for PTC development. As previously mentioned, Zhou et al. in their meta-analysis, concluded the association between rs2241766 and cancer incidence, while Hu et al. did not find similar results on the relationship between rs2241766 and breast cancer risk [26, 27]. In their study, Li et al. also found no association between this polymorphism and the risk of colorectal cancer [15]. In Mexico Mendez-Hernandez et al. showed that rs2241766 was associated with overweight and obesity in patients with breast cancer [28]. Cui et al., showed that the TT genotype and T allele frequencies of the rs2241766 polymorphism were observed in non-small-cell lung carcinoma patients compared to healthy controls. The results of the survival analysis showed that rs2241766 was significantly associated with the overall survival of patients with NSCLC after tumor removal [29]. Obesity, indicated by a high BMI, is believed to be associated with reduced cancer survival. However, some findings reinforce the belief of obesity paradox, so that some results indicate a poor prognosis in cancer patients with low BMI [30]. Neuhouser et al. evaluated the effect of weight gain on postmenopausal breast cancer risk in American women. They found that women who were overweight or obese were at an increased risk of developing the disease as compared with normal-weight women, and this risk was directly related to obesity [31]. Taghizadeh et al reported that obesity was significantly associated with an increased risk of death from prostate cancer. They also found that women with obesity had an increased risk for cancer death [32 ].

## Conflict of Interest:

We declare that we have no conflict of interest.
